# Imaging of MSC transplantation in neuroscience

**DOI:** 10.18632/oncotarget.14643

**Published:** 2017-01-13

**Authors:** Miriam Filippi, Marina Boido, Enzo Terreno

**Affiliations:** Molecular & Preclinical Imaging Centers, Department of Molecular Biotechnology and Health Sciences, University of Torino, Torino, Italy

**Keywords:** cellular imaging, mesenchymal stem cells, regenerative medicine, spinal cord injury, MRI, Neuroscience

Due to their stemness and unique paracrine, immunomodulatory and anti-inflammatory properties, Mesenchymal Stem Cells (MSCs) hold great promise for tissue regeneration and wound repair, as well as for the treatment of autoimmune disorders, tumors and cerebrovascular diseases. Notably, their relevance as potential therapeutic agents for nervous system diseases/lesions (including stroke, neurodegeneration, neuropathy, and traumatic damage) is also considerably increasing [1, 2]. Specifically, aiming at enhancing the cell/fiber rescue and the functional recovery, the MSC transplantation represents a promising therapeutic approach after serious traumatic events such as Spinal Cord Injury (SCI), that often lead to seriously disabling consequences [3]. Many key factors can influence the success of a cell therapy, including the origin of the cell population (allogenic *vs* autologous), the graft size, the administration route, the homing efficiency and the host environment. As a consequence, the opportunity to obtain spatial and temporal information on the graft distribution appears crucial [4]. The *in vivo* tracking of MSCs migration and biodistribution can be pursued using several clinical imaging techniques such as NIRF imaging, PAI, PET, SPECT, and MRI, and it is typically accomplished through the *in vitro* labelling of cells with a specific contrast agent. This method assures a non-invasive time-stratified analysis in living models, preserving the integrity of target organs and avoiding the animal sacrifice. Despite the good clinical potential showed by this approach, some common challenges remain, including the possibility of long-term monitoring, cytotoxicity, poor labeling efficiency, probe degradation, and difficult delivery of contrast to certain anatomic districts, such as the central nervous system (CNS).

As cell tracking experiments requires a good spatial resolution, MRI is certainly one of the preferred options. So far, the most used class of cell-labelling MRI agent is represented by the iron oxide nanoparticles (IONs) whose potential has been already demonstrated in some completed clinical trials aimed at tracking inflammatory or dendritic cells [5]. However, it has to be noticed that IONs-labeled cells appear as hypointense spots on T_2_- and T_2_^*^-weighted MR images, thus making sometimes difficult to discern them into anatomic areas with a low intrinsic MRI signal, as in micro-hemorrhage, or micro air bubbles commonly present after the surgery post-SCI. Moreover, besides being frequently affected by blooming artifacts, IONs are associated with dose-dependent cytotoxic phenomena, such that the high doses needed for successful labelling entail an acknowledged risk of compromising the cellular survival. These potential drawbacks can be overcome using the most commonly used class of clinical MRI agents based on paramagnetic Gd-based complexes. These chemicals produce bright spots on T_1_-weighted MRI and include molecules provided with extremely high thermodynamic and kinetic stability, hydrophilic character, small size, and biological inertness. Nevertheless, clinically-approved Gd-based agents cannot compete with IONs in terms of contrast detection sensitivity, and they still represent a challenging implementation in the field of imaging MSCs graft.

**Figure:**
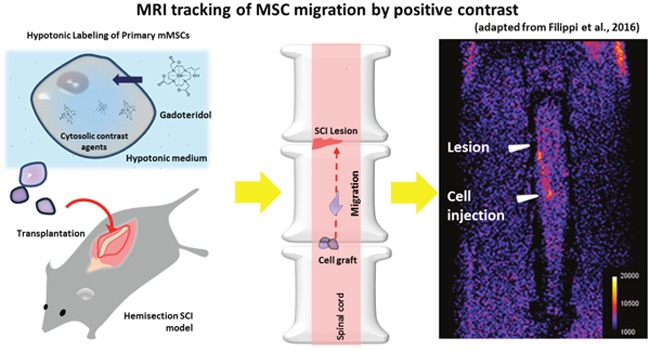


An important step forward to bridge the gap between Gd-based agents and IONs for *in vivo* cellular imaging has been recently achieved, allowing the cytosolic entrapment of high amount of the clinically approved MRI agent Gadoteridol (marketed as ProHance™) through a novel cell labelling procedure based on osmotic shock [6]. The escape from the endocytic internalization pathway is very crucial for enhancing the sensitivity in the detection of the bright contrast because the endosomal entrapment of the labelling agent is inevitably accompanied by a “quenching” of the T_1_ contrast [6]. Based on our experience, we argued that this labelling procedure could be helpful in tracking the migration of “therapeutic” MSCs in a murine SCI model (hemisection) [7]. Indeed, in addition to ascertain improvements in both cellular uptake and sensitivity in contrast detection *in vitro*, we found that the monitoring of MSCs migratory dynamics *in vivo* was feasible for about 10 days. By implanting MSCs about 1 mm caudally to spinal lesion site (as shown in the Figure below), it was possible to observe a migratory stream of cells progressively reaching the injured area in about 7 days. Moreover, the absence of alterations in the biological and functional profile of MSCs after the labeling step was verified *in vitro*, and confirmed *in vivo* by the evident motor recovery shown by treated animals, thus indicating the unaltered therapeutic efficacy of cells. In the context of experimental research, this efficient, safe, reliable, and simple labeling technique may serve to (*i*) test and compare different transplantation protocols, (*ii*) dynamically assess cell distribution, (*iii*) correlate the beneficial effects of the cell graft (estimated by behavioral tests) to the MRI observations, and (*iv*) analyze the ability of the injected cells to move in response to chemotactic stimuli *in vivo* and in real-time. Due to the relatively limited persistence of the contrast, the translational significance of such technique appears restricted to the early assessment of cell survival during the first days after injection, whose relevance however should not be underestimated considering that the detrimental response of the host CNS to the graft can determine death of exogenous cells within the first hours after transplantation [8].

In conclusion, the hypotonic labeling with positive MRI CAs represents a significant technical improvement in preclinical imaging of MSCs as applied to murine SCI, being potentially valid also in larger animals and humans, and lays the basis to address the current challenges imposed by the complexity of the CNS and the specific medical requirements of the stem cell-based therapies.
